# ZNF131 suppresses centrosome fragmentation in glioblastoma stem-like cells through regulation of HAUS5

**DOI:** 10.18632/oncotarget.18153

**Published:** 2017-05-24

**Authors:** Yu Ding, Jacob A. Herman, Chad M. Toledo, Jackie M. Lang, Philip Corrin, Emily J. Girard, Ryan Basom, Jeffrey J. Delrow, James M. Olson, Patrick J. Paddison

**Affiliations:** ^1^ Human Biology Division, Fred Hutchinson Cancer Research Center, Seattle, WA, USA; ^2^ Basic Sciences Division, Fred Hutchinson Cancer Research Center, Seattle, WA, USA; ^3^ Molecular and Cellular Biology Program, University of Washington, Seattle, WA, USA; ^4^ Clinical Research Division, Fred Hutchinson Cancer Research Center, Seattle, WA, USA; ^5^ Genomics and Bioinformatics Shared Resources, Fred Hutchinson Cancer Research Center, Seattle, WA, USA; ^6^ Novartis Institute for Biomedical Research, Shanghai, China; ^7^ Nurix Inc., San Francisco, CA, USA

**Keywords:** glioblastoma, ZNF131, HAUS5, Augmin/HAUS complex, cancer therapeutics

## Abstract

Zinc finger domain genes comprise ∼3% of the human genome, yet many of their functions remain unknown. Here we investigated roles for the vertebrate-specific BTB domain zinc finger gene *ZNF131* in the context of human brain tumors. We report that *ZNF131* is broadly required for Glioblastoma stem-like cell (GSC) viability, but dispensable for neural progenitor cell (NPC) viability. Examination of gene expression changes after *ZNF131* knockdown (kd) revealed that *ZNF131* activity notably promotes expression of Joubert Syndrome ciliopathy genes, including *KIF7*, *NPHP1*, and *TMEM237*, as well as *HAUS5*, a component of Augmin/HAUS complex that facilitates microtubule nucleation along the mitotic spindle. Of these genes only kd of *HAUS5* displayed GSC-specific viability loss. Critically, *HAUS5* ectopic expression was sufficient to suppress viability defects of *ZNF131* kd cells. Moreover, *ZNF131* and *HAUS5 kd* phenocopied each other in GSCs, each causing: mitotic arrest, centrosome fragmentation, loss of Augmin/HAUS complex on the mitotic spindle, and loss of GSC self-renewal and tumor formation capacity. In control NPCs, we observed centrosome fragmentation and lethality only when *HAUS5* kd was combined with kd of *HAUS*2 or *HAUS4*, demonstrating that the complex is essential in NPCs, but that GSCs have heightened requirement. Our results suggest that GSCs differentially rely on *ZNF131*-dependent expression of *HAUS5* as well as the Augmin/HAUS complex activity to maintain the integrity of centrosome function and viability.

## INTRODUCTION

Glioblastoma (GBM) is the most aggressive and common form of brain cancer in adults [1, 2]. Both adult and pediatric gliomas and other primary brain tumors appear to be hierarchically organized suggestive of a cancer stem cell origin [3–6]. Consistent with this notion, GBM stem-like cells (GSCs) have been isolated that retain the development potential and specific genetic alterations found in the patient's tumor [3, 4, 7, 8].

To identify new GBM therapeutic targets, we have previously performed shRNA screens in patient-derived GSC isolates and human neural progenitor cells (NPCs) for genes required for their *in vitro* expansion [9]. By carrying out control screens in proliferating fetal NPCs, which have similar expression profiles and developmental potential but are not transformed [7, 8], candidate GSC-specific therapeutic targets can be identified [9–11]. Further, by identifying cancer-lethal targets which cross validate in different GSC isolates that contain diverse cancer drivers, cancer therapeutic targets can be identified which may transcend tumor heterogeneity. Here, we validate one such candidate GSC-lethal gene, the putative transcription factor *ZNF131* and investigate its GSC-relevant function.

## RESULTS

### *ZNF131* retests as a GSC-lethal screen hits from genome-wide screens in GBM patient isolates

**Figure 1:**
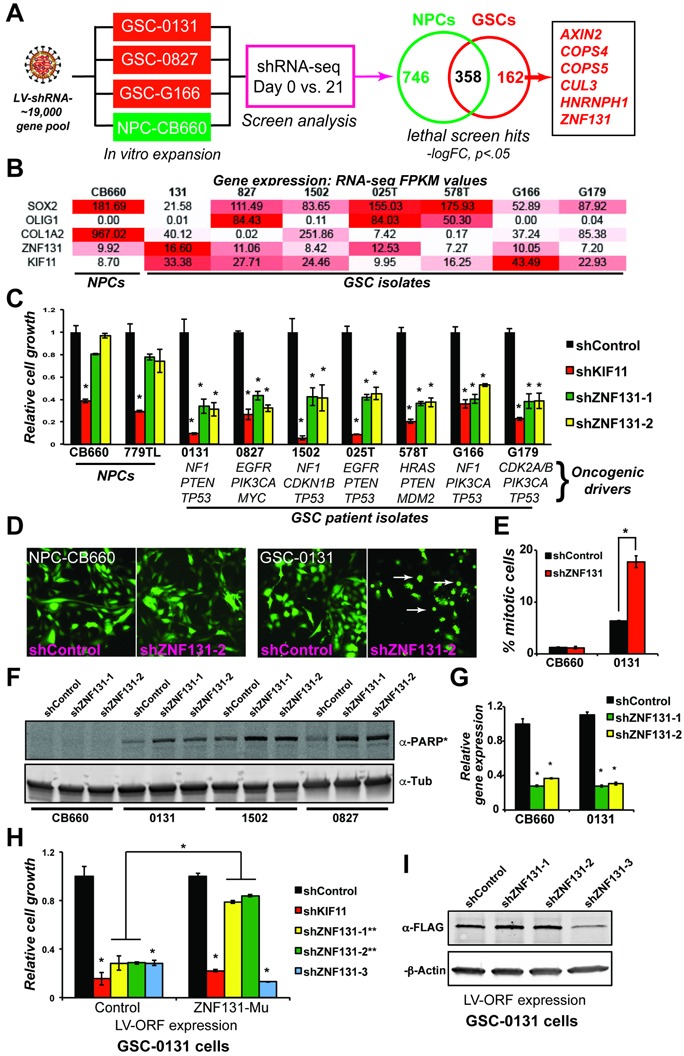
*ZNF131* is a candidate GSC-lethal gene **A**. Overview of GSCs and NPCs shRNA screens that gave rise to *ZNF131* as a candidate GSC-lethal gene. **B**. *ZNF131* expression among NPC and GSC isolates. Values are from FPKM normalized RNA-seq data from self-renewing *in vitro* cultures. **C**. Short term growth assays showing differential viability requirement for *ZNF131* among GSC and NPC isolates. Cell growth assays were performed 7 days after selection for LV-shRNAs in monolayer culture (*n* ≥ 3). **D**. Fluorescent micrographs of shRNA transduced cells (GFP+). Arrows show GSC cells displaying mitotic arrest phenotype observed with *ZNF131* kd. **E**. Quantification of mitotic cells from **D**.. **F**. Western blot to detect cleavage of PARP protein, an indicator of apoptotic cell death, in NPC-CB660 and three GSC isolates after knockdown of *ZNF131*. Cells were assayed 48hrs post-selection after LV-shRNA infection (*n* = 3). **G**. RT-qPCR analysis of *ZNF131* kd in NPCs and GSCs. Cells were assayed 48hrs post-selection after LV-shRNA infection (*n* = 3). **H**. Suppression of growth defects caused by shZNF131 using shRNA resistant *ZNF131* ORF. Cells were first infected with LV containing control or shRNA resistant ORF followed by LV-shRNA and assayed for cell growth in monolayer culture 7 days post selection (*n* = 3). Target sites for ZNF131 shRNAs #1 and #2 where both mutated in the ORF construct and thus made resistant, while the site for shRNA #3 was left unchanged. **I**. Western blots of shRNA resistant ORF expression. *indicates *p* < .01 student's *t*-test.

We have previously performed shRNA screens in three patient-derived GSC isolates, including, G166, 0131, and 0827 cells, and a control NPC isolate (CB660 cells [12]), for genes required for *in vitro* expansion under self-renewal conditions during monolayer outgrowth [9] (Figure 1A). By comparing GSC and NPC screen results, a list of 162 GSC-specific genes was produced that scored in at least two of the GSC screens, but not NPCs. We initially retested nine genes, six of which retested as being differentially required for GSC *in vitro* expansion (Figure 1A). Among these was *ZNF131*, which encodes a novel BTB domain zinc finger protein and putative transcription factor [13–16] that appears unique to vertebrates (according to the HomoloGene and UniGene databases). Since very little is known about *ZNF131* function, we decided to further characterize its role in promoting GSC self-renewal.

We first examined steady-state *ZNF131* expression levels in NPCs and GSCs. Figure 1B shows that *ZNF131* is robustly expressed in both NPCs and GSCs in a manner independent of GBM subtype (Figure 1B). We next examined the impact of *ZNF131* knockdown (kd) on GSC and NPC expansion using multiple GBM patient isolates. The results were consistent with *ZNF131* kd being generally lethal to GSCs regardless of specific genetic alterations (which were determined by exome-seq and CNV analysis (Supplementary Table S1)). We observed that *ZNF131* kd scored similar to an shRNA targeting *KIF11* in 7 out of 7 GSCs isolates examined (Figure 1C). *KIF11*, which encodes a microtubule motor protein required for mitotic progression in proliferating mammalian cells [17] and also GSC maintenance [18], serves as both a knockdown and proliferation control in our experiments. In contrast to GSCs, *ZNF131* kd in two different NPC isolates failed to produce a significant effect (Figure 1C).

Visual inspection of GSCs experiencing *ZNF131* kd revealed significant increases in mitotic cells, consistent with its knockdown causing mitotic arrest or catastrophe (Figures 1D & 1E). Similar phenotypes were observed with all three shZNF131s examined (not shown; see below). In addition, we observed dramatic induction of apoptosis in three GSC isolates examined after *ZNF131* kd, including adult [0131 & 0827] and pediatric [1502] isolates, but not NPCs (Figure 1F), again suggesting GSC-specific requirement.

Examination of *ZNF131* kd in GSCs and NPCs demonstrated similar robust silencing by two independent shRNAs, suggesting that the observed differences were not due to poor silencing in NPCs (Figure 1G). Moreover, to ensure that the results were due to on-target effects, we performed complementation studies using a mutated *ZNF131* ORF resistant to 2 out of 3 effective *ZNF131* shRNAs (Materials and Methods) (Figures 1H & 1I). For these experiments, cells were first infected with LV containing control or shRNA-resistant-ORF followed by LV-shControl, shKIF11, or shZNF131 and assayed for cell growth. Target sites for ZNF131 shRNAs #1 and #2 where both mutated in the ORF construct and thus made resistant, while the site for shRNA #3 was left unchanged. Expressing this mutant ORF in GSC-0131 cells dramatically rescued lethal effects of *ZNF131* kd (Figures 1H & 1I), demonstrating that the lethality is due to targeting of *ZNF131* and not due to an off-target effect.

### *ZNF131* is required for maintaining expression of *HAUS5* in GBM stem-like cells

*ZNF131* encodes a putative transcriptional regulator [13–16] predominantly expressed in the developing central nervous system and adult brain, testis, and thymus [15]. Despite strong suggestive evidence that ZNF131 is a transcription factor from *in vitro* and cell-based reporter assays [13–16] [19] [20], no direct transcriptional regulatory targets of ZNF131 have been identified in cells. And while two studies have suggested ZNF131 transcriptional activities are coordinated with binding partners such as Kaiso/ZBTB33 [19] and human polycomb protein 2 [20], other studies suggest that at least Kaiso functions independently of ZNF131 [21]. Thus, *ZNF131*'s function(s) remains unclear.

**Figure 2:**
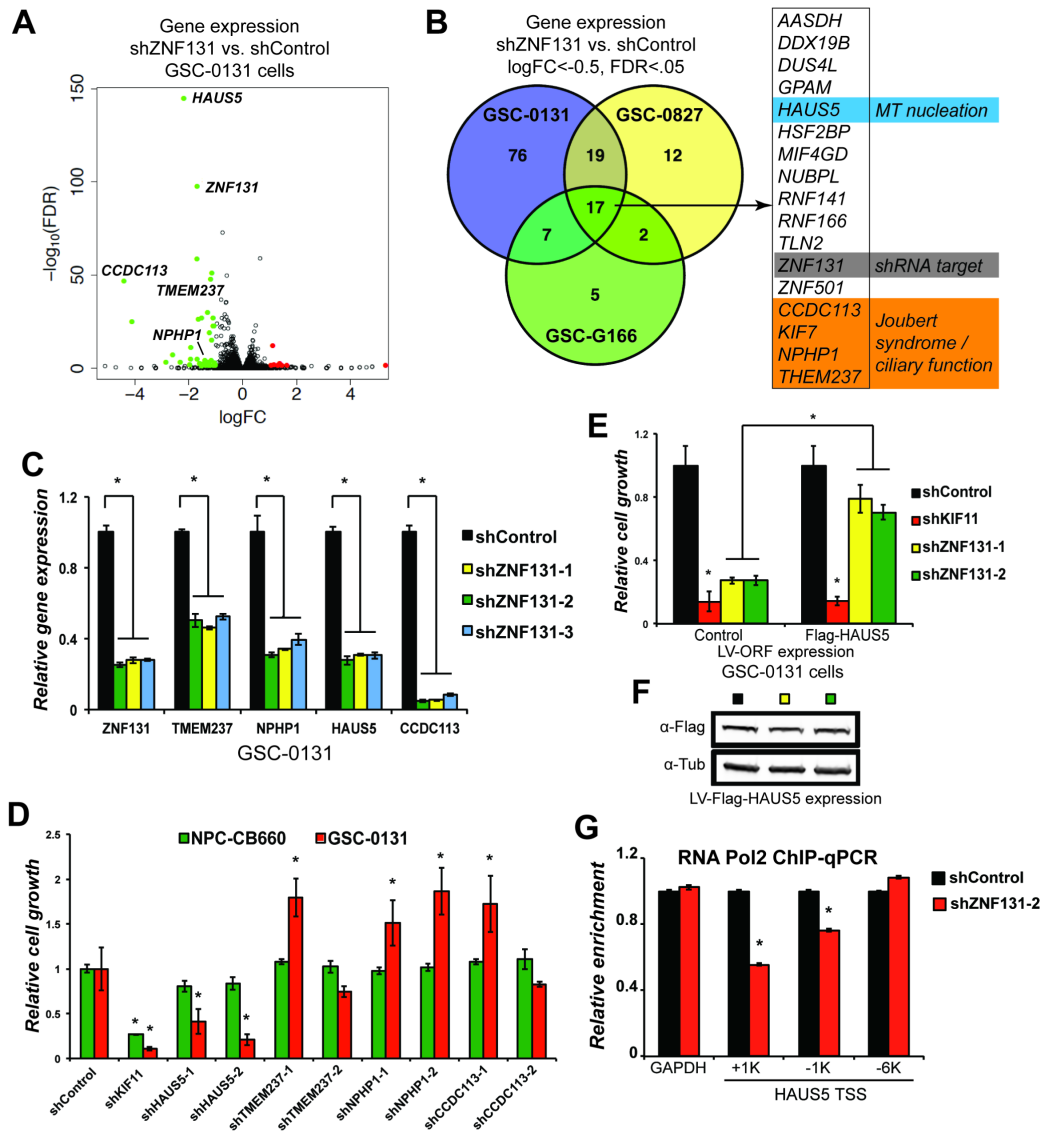
ZNF131 regulates expression of the *HAUS5* gene to promote expansion of GSCs **A**. A volcano plot of changes in mRNA-sequencing resulting from *ZNF131* kd in GSC-0131 cells. **B**. A Venn diagram summary of overlap in genes whose expression is down regulated after *ZNF131* kd in 0131, 0827, and G166 GSC isolates. RNA-seq data is available in Supplementary Table S2. **C**. RT-qPCR confirmation of RNA-seq results from **A**. and **B**. for *ZNF131*, *TMEM237*, *NPHP1*, *HAUS5*, and *CCDC113*. **D**. Retests of a subset of genes from **B**. in knockdown growth assays in GSC-0131 and NPC-CB660 cells, including for *ZNF131*, *TMEM237*, *NPHP1*, *HAUS5*, and *CCDC113*. **E**. Suppression of *ZNF131* kd lethality by stable ectopic expression of the *HAUS5* ORF. Cells were first infected with LV containing control or *HAUS5* ORF followed by LV-shRNA and assayed for cell growth in monolayer culture 7 days post selection (*n* = 3). **F**. Western blot analysis of GSC-0131 cells expressing *HAUS5* ORF used for **E**.. **G**. Chromatin-immunoprecipitation of RNA polymerase II at the *HAUS5* or *GAPDH* (control) promoter with and without *ZNF131* kd. *indicates *p* < .01 student's *t*-test.

To further characterize *ZNF131*, we first confirmed its cellular localization as exclusively nuclear in GBM cells (Supplementary Figure S1), consistent with previous studies [19, 20]. Next, we performed gene expression profiling *via* mRNA-sequencing after knockdown of *ZNF131* in three different GSC isolates (G166, 0131, and 0827 cells) during self-renewal conditions. The goal of these experiments was to identify indirect or direct transcriptional downstream target(s) responsible for maintenance of GBM cell viability. Analyzing RNA-seq results from shControl *versus* shZNF131 treated cells yielded a surprisingly small set of genes whose expression changed in response to *ZNF131* kd (Figures 2A & 2B). Using a cut off of |logFC| ≥ 0.585, FDR < 5% (Material and Methods), *ZNF131* kd altered expression of only 107, 48, and 27 genes in GSC 0131, 0827, and G166 isolates, respectively (Supplementary Table S2). The majority of affected genes exhibited loss of expression and there were 17 genes consistently down regulated between 3 different GSC isolates examined (Figure 2B; Supplementary Table S2). Intriguingly, gene set enrichment analysis for these 17 genes revealed that involvement in “Joubert Syndrome and Related Disorders” (JSRDs) (*p* = 1.487E-7) for *KIF7*, *NPHP1*, and *TMEM237* [22]. Expanding the GSEA to include all up or down regulated genes also produced significant enrichment for proteins in the centrosome-cilium interface (Supplementary Figures S2A & S2B).

JSRDs represent a group of physiological and developmental disorders, including neurodevelopmental disorders, caused by dysfunction of the primary cilia, which plays key roles in sensory perception and also developmental signaling [23]. Further examination of the list revealed *CCDC113*, which codes for a centrosome-associated protein involved in formation of the primary cilium [24], and *HAUS5*, which encodes a member of the Augmin/HAUS complex and participates in nucleation of microtubules along the mitotic spindle [25–27] (Figures 2A & 2B). Because we observed GSC-specific mitotic arrest after *ZNF131* kd and cilium function, centrosome duplication, and mitotic spindle formation are inter-related [28], we wondered whether one or more of these genes are responsible for *ZNF131* kd loss viability in GSCs. Thereby, we confirmed *ZNF131*-dependent regulation of these genes using three independent *ZNF131* shRNAs, all of which replicated RNA-seq results and indicated that their change in mRNA levels is not due to an off-target effect (Figure 1C). We also confirmed the effect in NPCs (Supplementary Figure S3A). We then examined effects of kd on stem cell expansion. Only kd of *HAUS5* showed GSC-specific lethality similar to *ZNF131* kd (Figure 2D). In contrast, kd of *CCDC113*, *NPHP1*, and *TMEM237* triggered GSC-specific enhancements of growth, possibly suggesting that primary cilium functions to limit their *in vitro* expansion.

This suggested that *HAUS5* could be the relevant regulatory target of ZNF131 for maintaining GSC viability. To demonstrate this phenotypically, we performed complementation experiments. Figures 2E and 2F shows that the effects of *ZNF131* kd are dramatically reversed in GSC-0131 cells ectopically expressing the *HAUS5* ORF. Importantly, *HAUS5* expression did not suppress the growth inhibitory effects of *KIF11* kd, indicating the effects are specific to *ZNF131* activity. Further confirmatory experiments in GSCs and GSC-tumors are presented below in Figure 7.

We further examined whether loss of *HAUS5* mRNA levels resulting from *ZNF131* kd is consistent with transcriptional regulation. To this end, we examined levels of RNA polymerase (RNAP) at the several positions around the *HAUS5* transcription start site (TSS) and also in a control gene, *GAPDH*, whose expression was not changed by *ZNF131* kd. Figure 2G shows that RNAP levels drop around the *HAUS5* TSS and in its gene body after *ZNF131* kd, but remain constant at a distal site (−6 kb from TSS) and at the GAPDH promoter. This suggests that ZNF131 activity promotes RNAP recruitment or helps maintain RNAP steady state levels at the *HAUS5* promoter. (Unfortunately, we will unable to perform endogenous ZNF131-ChIP experiments at these same promoters due to lack of suitable commercial ZNF131 antibodies.) Further, although endogenous targets of ZNF131 have not previously been identified, an *in vitro* DNA binding site screen revealed a 12bp palindrome sequence GTCGCR-(X)n-YGCGAC as potential binding motif [13]. The *HAUS5* locus does have partial matches for this sequence, GTCGC-(X)n-GCGAC, at TSS-20bp and TSS+851bp. Regardless, these results demonstrate that ZNF131 expression controls steady state levels of *HAUS5* mRNA and that this regulation is sufficient to explain ZNF131's GSC-specific requirement.

To find further support of this notion, we examined the co-expression of *ZNF131* and *HAUS5* in primary human NPCs and astrocytes and 22 GSC isolates (using data from [29] which includes all of the GSCs used in Figure 1C)(Supplementary Figures 3B & 3C). The results reveal that *ZNF131* and *HAUS5* significantly co-vary across samples, with a trend toward higher expression of both *ZNF131* and *HAUS5* in GSCs. This further suggests that the genes are transcriptionally linked.

### *ZNF131* loss in GSCs, but not NPCs, results in centrosome fragmentation and loss of spindle-associated MT nucleation

The HAUS5 protein is part of an 8-subunit complex Augmin/HAUS complex that has been shown to play critical roles in spindle microtubule (MT) generation Drosophila S2 cells and HeLa cells [25–27]. During mitosis, the mitotic spindle is erected *via* MT nucleation from centrosomes (i.e., spindle poles) and also along the emerging spindle at chromosome proximal regions [31]. The predominant MT nucleation sites are the centrosomes, where γ-tubulin ring complex (γ-TuRC) acts as the major MT nucleation complex [31]. By contrast, the Augmin/HAUS complex is responsible for centrosome-independent, spindle-based MT nucleation through recruitment of γ-TuRC to pre-existing MTs [25–27].

Augmin/HAUS complex members were originally identified from an RNAi screen in Drosophila S2 cells identifying genes required for localization of γ-tubulin along the spindle but not the centrosome [27]. Knockdown of complex members led to reduction in over all spindle MT number, causing relatively mild perturbations in spindle function [27]. However, in combination with kd of genes required for centrosome-specific γ-TuRC recruitment, disruptions in spindle bipolarity and chromosome alignment were more dramatic [27]. Characterization of human orthologs of the complex in HeLa cells, revealed similar, albeit more severe, phenotypes after knockdown of each Augmin/HAUS component [25, 26], including centrosome fragmentation and multipolar spindles. Thus, Augmin/HAUS is needed to support construction of the mitotic spindle and, as a result, proper kinetochore-MT attachments.

**Figure 3:**
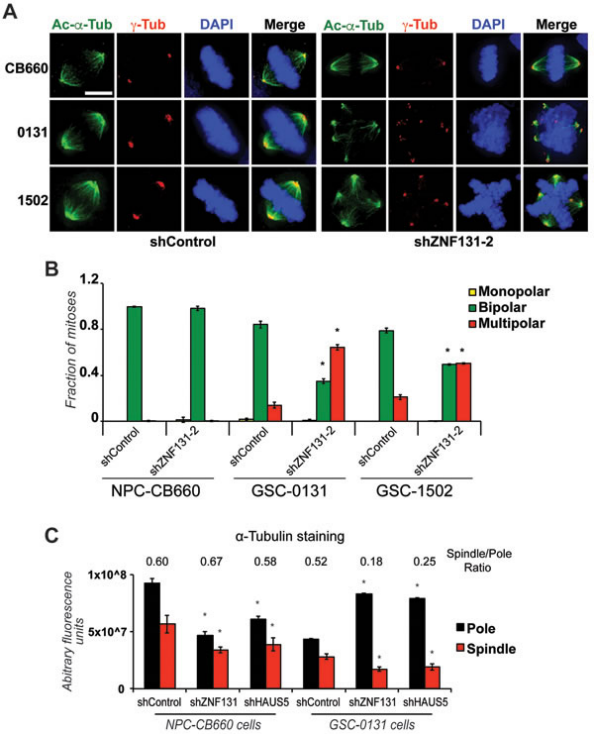
*ZNF131* kd causes centrosome fragmentation, multipolar spindle formation, and loss of MT-nucleation **A**. Examination of spindle polarity and centrosome morphology in NPCs and GSCs with and without *ZNF131* kd. Cells were stained for Acetylated-α-Tubulin and γ-Tubulin to reveal changes to spindle and centrosome, respectively, and analyzed using deconvolved 3D images from z-stacked sections (0.2μm intervals). Scale bar indicates 5 μm. **B**. Quantification of spindle polarity in **A**. (*n* > 20). **C**. Quantification of α-tubulin staining at spindle pole and along spindle in NPCs and GSCs with and without *ZNF131* and *HAUS5* kd (*n* = 5).

To further investigate the relationship between ZNF131 and HAUS5 activity, we first examined spindle bipolarity after their kd (Figures 3A & 3B; Supplementary Figures S4A-S4C). We stained cells for γ-tubulin, which is concentrated at the centrosome during mitosis [32], and α-tubulin, which allows visualization of the entire mitotic spindle. This revealed that *ZNF131* kd produces a high proportion of GSC cells (> 50%) with multipolar spindles and with concentrated γ-tubulin staining at their ends, without affecting NPC mitoses (Figures 3A & 3B). Staining with pericentrin, which interacts with and likely recruits γ-tubulin to centrosomes [33], produced nearly identical results, as did kd of *HAUS5* (Supplementary Figures S4A & S2B). Importantly, similar phenotypes were produced with three different ZNF131 shRNAs (Supplementary Figure 5). Thus, these results are consistent with *ZNF131* and *HAUS5* kd causing centrosome fragmentation in GSCs.

Since the HAUS5 is presumably rate-limiting for MT-nucleation along the mitotic spindle, we next assayed MT density along the spindle and at the spindle pole, as measured by α-tubulin intensity, in GSCs and NPCs with and without *ZNF131* and *HAUS5* kd. In NPCs, the results indicated that kd of *ZNF131* and *HAUS5* results in nearly identical reduction of α-tubulin intensity at both the spindle poles and along the spindle, without resulting in dramatic changes spindle *versus* pole α-tubulin intensity ratios (Figure 3C). This suggests that there is a significant, general loss of MT-nucleation at the spindle in NPCs, which does not significantly impact spindle polarity or viability. By contrast, in GSCs both *ZNF131* and *HAUS5* loss resulted in dramatic increases in α-tubulin intensities around poles and dramatic loss along the spindle (Figure 3C), skewing intensity ratios, consistent with loss of MT nucleation along the spindle. It is conceivable that the dramatic increases in pole-associated MT staining reflects alterations in feed back regulation of MT nucleation (i.e., γ-TuRC recruitment).

To further investigate consequences of ZNF131 kd, we examined alterations in centrosome size in GSCs and NPCs. To this end, centrosome size was determined by examining the volume of centrosomes based on Pericentrin staining [34] (Supplementary Figures S6A & S6B). This analysis revealed, first, that GSCs have larger centrosomes compared to NPCs, consistent with centrosome alteration and, second, that loss of *ZNF131* causes dramatic reduction in centrosome volume only in GSCs, consistent with centrosome fragmentation (Supplementary Figures S6A). Remarkably, this effect was reversed by ectopic expression of *HAUS5* in GSC-0131 cells (Supplementary Figures S6B), further demonstrating that HAUS5 activity is down stream of ZNF131 function. These results are consistent with the notion that ZNF131-dependent regulation of *HAUS5* expression is critical in GSCs, but not NPCs, for maintaining integrity of centrosome function and spindle bi-polarity during mitosis.

### *ZNF131* inhibition results in loss of Augmin/HAUS complex along the mitotic spindle

**Figure 4:**
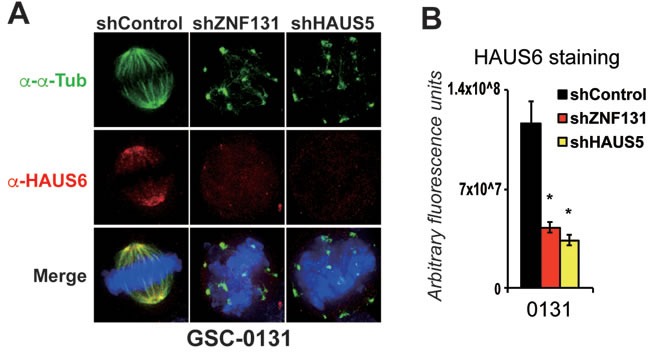
Knockdown of *ZNF131* and *HAUS5* results in loss of Augmin/HAUS complex along the mitotic spindle **A**. HAUS6 staining along the mitotic spindle of GSCs and NPCs with and without *ZNF131* and *HAUS5* kd. **B**. Quantification of **A**. using deconvolved 3D images from z-stacked sections (0.2μm intervals) as detailed in methods details. *indicates *p* < .01 student's *t*-test.

To further ensure that *ZNF131* kd was impacting Augmin/HAUS complex, we examined staining of HAUS6, which has been previously used assess human Augmin/HAUS complex abundance on along the mitotic spindle [26] (Figures 4A & 4B). Again, *ZNF131* and *HAUS5* kd phenocopied each other, producing indistinguishable results, showing profound loss of HAUS6 staining along the entire mitotic spindle. GSC-0131 cells showed almost a 4-fold reduction in HAUS6 staining (Figure 4B). Taken together, these results demonstrate that *ZNF131* or *HAUS5* inhibition similarly result in GSC-specific centrosome fragmentation, loss of spindle bipolarity, loss of spindle-associated MT nucleation, and loss of Augmin/HAUS complex along the mitotic spindle.

### *ZNF131* or *HAUS5* over-expression stimulates microtubule nucleation in GSCs

**Figure 5:**
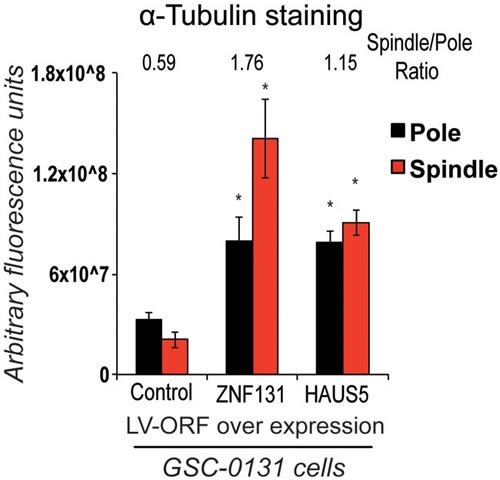
Overexpression of either ZNF131 or HAUS5 kd cause increased MT-nucleation along the mitotic spindle and at spindle poles Quantification of α-tubulin staining at spindle pole and along spindle was performed 48hrs after selection for LV-ZNF131 and LV-HAUS5 in GSC-0131 cells as detailed in methods (*n* = 30 for each group) (using devolved 3D images aquired by a Deltavision imaging station). *indicates *p*-value < .001, as compared to control values.

One question arising from the above studies is why ZNF131 would only regulate one member of the Augmin complex, especially if other members of the complex appear to make equal contributions to promoting activity [26]. This would seem an inefficient form of regulation unless modulation of expression of *HAUS5* is sufficient to affect the overall activity of the Augmin/HAUS complex. To address this question, we examined the changes in MT density along the spindle in GSC-0131 cells with and without ectopic expression of *ZNF131* and *HAUS5*. Remarkably, ectopic expression of either *ZNF131* or *HAUS5* in GSC-0131 cells dramatically increased MT density both near the spindle poles and along the spindle between 2-3-fold (Figure 5). This suggests that ZNF131 and HAUS5 activity are rate limiting for MT-nucleation in GSCs.

### The Augmin/HAUS complex is essential for suppressing centrosome fragmentation and promoting spindle bipolarity in NPCs

Two questions arising from the above studies include whether the Augmin/HAUS complex is indeed essential for mitosis in non-neoplastic cells and whether the centrosome fragmentation phenotypes require the presence of supernumerary centrosomes (i.e., centrosome amplification) [35]. To address these questions, we used NPCs, since they are diploid and do not have supernumerary centrosomes as judged by pericentrin and γ-tubulin staining. Since kd of *HAUS5* has little affect on viability or mitosis of NPCs, we performed combination kd studies of Augmin/HAUS complex members. To this end, we first examined differential effect of kd of single Augmin/HAUS complex members in GSCs and NPCs. We found that *HAUS2* and *HAUS4* kd showed GSC-specific growth defects similar to *HAUS5* kd; kd of *HAUS1* and *HAUS8* had no effect on GSCs or NPCs; and that *HAUS6* kd was growth inhibitory to both GSCs and NPCs (not shown). The latter result suggested that the Augmin/HAUS complex could be essential in NPCs.

**Figure 6:**
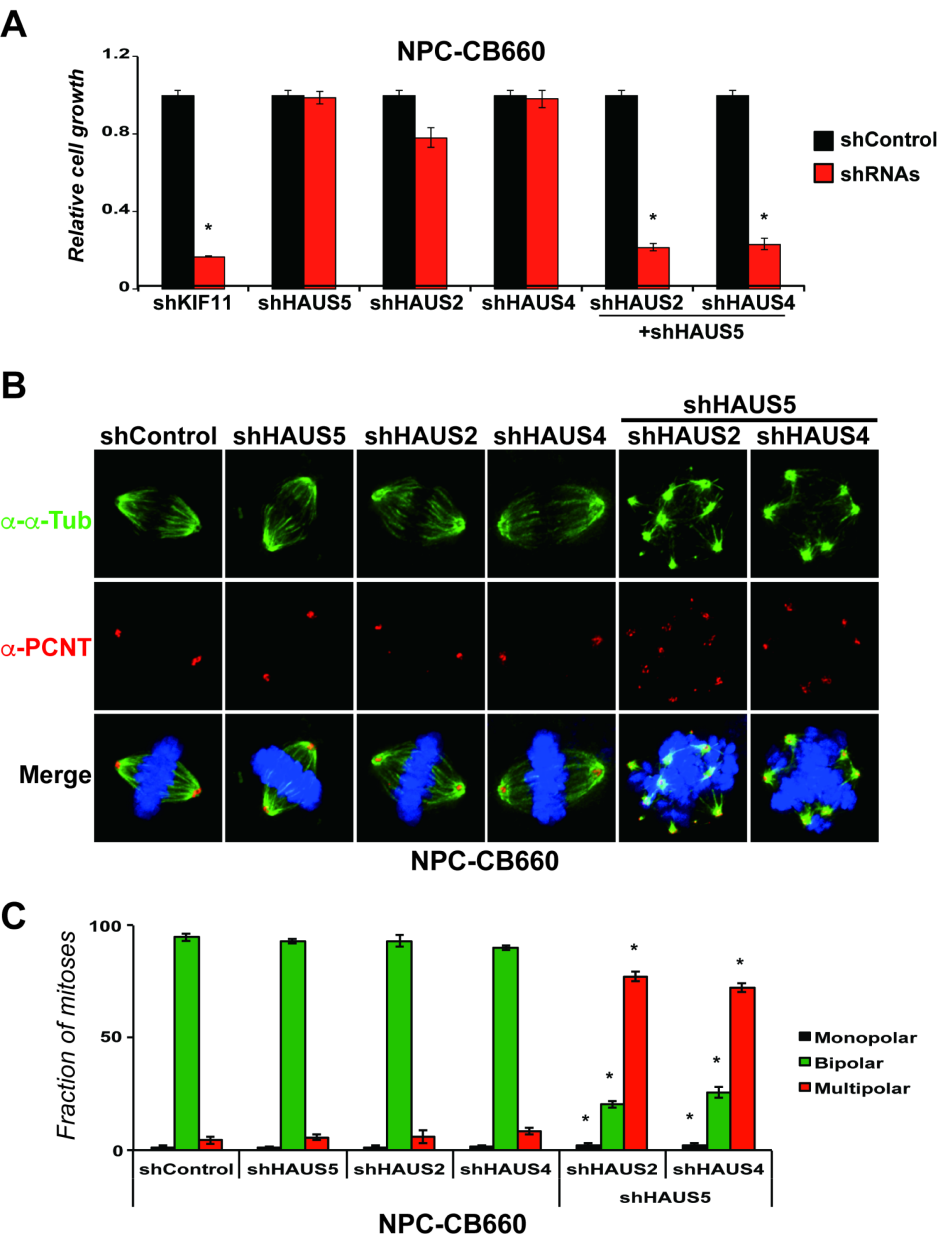
Combinatorial kd of HAUS5+HAUS2 or HAUS5+HAUS4 cause centrosome fragmentation and multipolar spindle formation in NPC-CB660 cells **A**. Combination of HAUS5+HAUS2 or HAUS5+HAUS4 kd are synthetic lethal in NSC-CB660 cells, based on growth assays 7 days post selection with LV-shRNAs in monolayer culture. Experiments were performed as detailed in Methods (*n* = 3) using dual expression shRNA LV vectors. **B**. Representative images of of NSC-CB660 cells after knockdown of HAUS2, HAUS4, and/or HAUS5. Cells were stained for pericentrin and tubulin 48hrs after selection for LV-shRNAs according to Methods. **C**. Examination of spindle polarity from **B**. (*n* > 20). *indicates *p*-value < .01.

To further examine this in the context of *HAUS5* function, we combined kd of *HAUS5* with kd of *HAUS2* or *HAUS4* in NPCs (Figure 6). We find that either combination of *HAUS5+HAUS2* or *HAUS5+HAUS4* kd are synthetic lethal in NPCs, producing similar growth inhibition to *KIF11* kd when combined, but no significant affect alone (Figure 6A). We then examined centrosome morphology and spindle bipolarity in these cells. The results exactly mirror the effects of kd of *HAUS5* alone in the GSCs. *HAUS5+HAUS2* or *HAUS5+HAUS4* kd resulted in fragmented centrosomes and multipolar spindles in the majority of cells (> 70%), without noticeable perturbation in single gene kd cells (Figures 6B & 6C).

To ensure that these results were not simply the result of differences in expression of different HAUS complex members (for example, perhaps NPCs have more expression and GSCs less), we compared gene expression of the HAUS1-8 genes in NPCs and GSCs used for viability experiments in Figure 1C (Supplementary Figure 7). The results did not show discernable differences, which would explain GSC-specific sensitivity or NPC-specific resistance to their inhibition.

These results demonstrate that the Augmin/HAUS complex is essential for growth of diploid NPCs and that centrosome amplification is not a requirement for centrosome fragmentation resulting from loss of Augmin/HAUS complex activity.

### *ZNF131* and *HAUS5* knockdown cause similar effects on GSC expansion, self-renewal, and tumor formation

**Figure 7:**
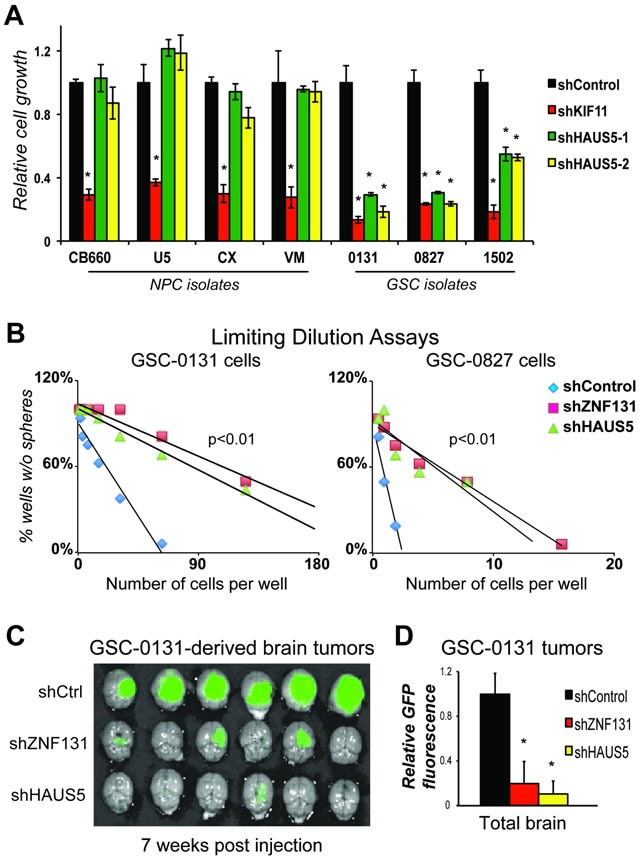
*HAUS5* kd phenocopies *ZNF131* kd in GSC growth, self-renewal, and tumorigenesis assays **A**. Short term growth assays showing differential viability requirement for *HAUS5* among GSC and NPC isolates, performed as in Figure 1 (*n* = 3). **B**. Tumorisphere limiting dilution assays with and without *ZNF131* and *HAUS5* kd in GSC-131 and GSC-0827 isolates. **C**. GSC-0131 brain tumorigenesis assays in immunocompromised mice with and without *ZNF131* and *HAUS5* kd in GSC-0131 (*n* = 6), seven weeks post-injection. **D**. Quantification of GFP intensity from (*n* = 6) **C**.. *indicates *p* < .01 student's *t*-test.

To further ensure that *HAUS5* kd phenocopies *ZNF131* kd, we examined the effects of *HAUS5* kd on cell expansion in four human NPC and three GSC isolates (Figure 7A). Similar to *ZNF131* kd, *HAUS5* kd results in significantly diminished expansion capacity similar to shKIF11 in GSCs, but has no effect on expansion of NPCs (Figure 7A). We next examined effects on tumorisphere formation using a limiting dilution assay, a surrogate assay for stem cell self-renewal [3, 30](Figure 7B). These studies revealed that *ZNF131* and *HAUS5* kd produce indistinguishable limiting dilution curves showing significant suppression of sphere formation capacity of GSC-0131 and GSC-0827 cells.

Similar results were also obtained by brain tumor formation assays in immunocomprised mice. Knockdown of *ZNF131* or *HAUS5* each caused similar, profound loss of tumor formation for GSC-0131-derived xenograft tumors, where tumors growth was assayed 7 weeks post-injection (Figures 7C & 7D). Thus, these results are consistent with the notion that among *HAUS5* kd replicates phenotypes produced by *ZNF131* loss in GSCs and that loss of their activity compromises GBM tumor formation.

## DISCUSSION

Here, we identify *ZNF131* as a gene differentially required for patient-derived GSC expansion and determined its primary GSC-relevant activity. We find that GSC-specific requirement for *ZNF131* activity arises from regulation of *HAUS5* gene expression. Expression of either *ZNF131* or *HAUS5* could complement the effect of *ZNF131* kd in GSCs. HAUS5 protein is a member of the 8 component Augmin/HAUS complex that stimulates MT nucleation along the mitotic spindle [26, 27]. In HeLa cells, the Augmin/HAUS complex is required for centrosome and spindle integrity [26]. In our GSC isolates, we find that partial loss of either *ZNF131* or *HAUS5* caused profound loss of the Augmin/HAUS complex from the spindle, centrosome fragmentation, and spindle multipolarity accompanied by loss of self-renewal and tumorigenesis capacity. However, their kd in NPCs produced little or know effects on spindle or centrosome integrity. Remarkably, we also found that over expression of either *ZNF131* or *HAUS5* could stimulate increases in MT density along the spindle, further suggesting that both genes are rate-limiting MT nucleation. Taken together, the results demonstrate a novel and unexpected role for ZNF131 activity in promoting GBM cell viability.

One critical question is whether ZNF131-dependent regulation of HAUS5 is an artifact caused by an off-target RNAi effect, whereby shRNAs designed to target *ZNF131* also bind to *HAUS5* mRNA. Off target effects of this nature are a cause of concern (e.g., [36]). However, we performed multiple independent experiments that demonstrate our phenotypes are not due to off target effects. First, multiple shRNAs were used to produce kd and phenotypic effects; and second, complementation studies were performed with RNAi resistant ORFs. Both of these controls demonstrated that ZNF131-specific effects cannot be due shZN131s targeting *HAUS5*.

Previous work has established that *ZNF131* encodes a novel BTB (brica-a-brac, tramtrack and broad complex) domain zinc finger protein and putative transcription factor [13–16]. Our results demonstrate the first down stream targets of ZNF131 in mammals, which include genes associated with Joubert Syndrome and primary cilium function and *HAUS5*. It is conceivable that *ZNF131* evolved as an adaption to promote or maintain Augmin/HAUS complex activity during key times in development *via* transcriptional regulation of *HAUS5*. We were able to show that ZNF131 affects steady-state levels of RNAP at that HAUS5 promoter, consistent with transcriptional regulation. However, we were unable to establish a mechanism for this effect (e.g., is ZNF131 acting as a sequence-specific DNA-binding transcription factor or is it acting through a more exotic mechanism to affect HAUS5 transcription?). Future work will be required to establish a precise mechanism for this regulation.

Another key question arising from this work is why GSC isolates are more sensitive to loss of ZNF131, HAUS5, and the Augmin/HAUS complex activity than NPCs. The 8-subunit complex Augmin/HAUS complex has been shown to play critical roles in spindle microtubule (MT) generation Drosophila S2 cells and HeLa cells [25–27]. During mitosis, the mitotic spindle is erected *via* MT nucleation at spindle poles but also along the emerging spindle at chromosome proximal regions [31]. While the γ-tubulin ring complex (γ-TuRC) acts as the major MT nucleation complex [31] at centrosomes, the Augmin/HAUS complex is responsible for centrosome-independent, spindle-based MT nucleation through recruitment of γ-TuRC to pre-existing MTs [25–27]. Knockdown of Augmin/HAUS complex members leads to reduction in over-all spindle MT number, causing relatively mild perturbations in spindle function [27]. However, in combination with kd of genes required for centrosome-specific γ-TuRC recruitment, disruptions in spindle bipolarity and chromosome alignment were more dramatic [27]. Knockdown of the human orthologs of the complex member genes in HeLa cells, revealed more severe perturbation of spindle function [25, 26]. From our results, in non-transformed neural progenitors, we find significant reductions in alpha-tubulin staining both at the spindle pole and along the spindle after ZNF131 and HAUS5 knockdown (Figure 3C), consistent with loss of MT number. However, these reductions do not lead to loss of spindle integrity (Figures 3A & 3B). In GSCs, both ZNF131 and HAUS5 knockdown also trigger dramatic changes in alpha-tubulin accumulation at the pole and along the spindle, although, in this case, pole accumulation significantly increased while accumulation along the spindle significantly decreased. (This may reflect inherent differences in regulation of the γ-TuRC MT complex at the spindle pole in GSCs.) However, in contrast to NPCs, GSCs displayed dramatic loss of spindle and centrosome integrity (Figures 3A & 3B; Supplementary Figure S6). We assert loss of spindle integrity is the primary cause of lethality after loss of ZNF131 or HAUS5 activity in GSCs.

There is growing evidence that most cancers have numerical or structural alterations in centrosomes [35]. In particular, centrosome amplification, or the presence of supernumerary centrioles caused by their over-replication, failed cytokinesis, or cell fusion, has been documented in multiple cancers [37–41]. Cancer cells compensate for centrosome amplification by “clustering” centrosomes into two poles, in part, *via* the activity of the kinesin protein Ncd/HSET [42]. Intriguingly, we were able to observe significant increases in centrosome volume in GSCs and observed centrosome fragmentation *ZNF131* or *HAUS5 kd* in GSCs*,* which is very similar to de-clustering phenotypes for cells with amplified centrosomes. However, we were also able to observe centrosome fragmentation in our NPCs, which are diploid and do not have centrosome amplification (as judged by γ-tubulin and pericentrin staining) with *HAUS5*+*HAUS2* or *HAUS5*+*HAUS4 kd.* Thus, it would follow that centrosome fragmentation can occur in the absence of centrosomes amplification when Augmin/HAUS complex activity levels are critically low. One possible explanation is that Augmin/HAUS-dependent MT nucleation helps balance forces applied on centrosomes as the spindle is erected, during or after centrosomes separate.

Pelletier and colleagues have suggested that Augmin/HAUS complex activities are opposed by the action of the nonmotor spindle protein NuMA [26]. NuMA plays a functional role in organizing microtubules of the mitotic spindle [43, 44]. Intriguingly, inhibition of NuMA can suppress defects in HAUS complex activity [26]. Thus, it is possible that NuMA activity could be higher in GSCs than NPCs, which could explain the difference in ZNF131 and HAUS complex requirement.

Another related possibility is that aneuploidy and genomic alterations have roles in inducing spindle stress (i.e., unbalancing forces) during mitotic spindle erection [45]. For example, it is has been suggested that capture of chromosomes in aneuploid cells can produce additional pulling forces sufficient to cause mother-daughter centrioles to loose integrity during prolonged mitotic delays [45]. In this scenario, ZNF131 and HAUS5 function could help compensate for chromosome alignment defects in GBM cells through maintaining sufficient levels of MT nucleation along the spindle. Supporting this general idea, we have previously reported that GBM cells have RTK-Ras induced chromosome alignment defects that cause added requirement for BuGZ and BubR1 [10, 11, 46]. However, additional studies focused on alterations of spindle forces in GSCs will be required to address these ideas.

Our studies suggest that the Augmin/HAUS complex activity may represent therapeutic target for GBM. Remarkably, targeting of ZNF131 and HAUS5 resulted in dramatic loss of viability in multiple GSC isolates (7 of 7) with diverse oncogenic drivers (Figure 1C), without significantly affecting NPCs. The fact that GSCs with different oncogenic drivers could be targeted may bode well for targeting GBM tumors, which are notoriously heterogeneous [47–49]. Their knockdown also dramatically reduced GSC self-renewal (i.e., tumor sphere formation) and the formation of GSC-derived tumors (Figure 7). Thus, the results may have relevance in actual tumors and could suggest a therapeutic window.

However, one related question is whether ZNF131, HAUS5, and the HAUS complex are essential genes. In addition to the synthetic lethal experiments shown in Figure 6, we recently performed a series of CRISPR-Cas9 knockout viability screens in both NPCs and GSCs (e.g., NPC-CB660, GSC-0131, and GSC-0827) [50]. In contrast to RNAi-knockdown screens, which tend to induce hypomorphic gene activities (i.e., partial loss of function), CRISPR-Cas9-based screens can generate highly penetrate insertion-deletion mutations that effectively knockout protein function [51, 52]. Critically, from our CRISPR-Cas9 screens we observe that sgRNAs targeting ZNF131 and HAUS complex members (HAUS2, HAUS3, HAUS4, HAUS5, HAUS7, and HAUS8) all scored as essential (being depleted over time during expansion) in both NPCs and GSCs. This is consistent with the notion that the GSC-specific differences presented here are the result of partial loss of gene activities that can be tolerated in NPCs but not GSCs. This certainly does not disqualify the Augmin/HAUS complex as a key GBM target. In fact, it is conceivable that MT nucleation inhibitors could have a better therapeutic window than other spindle targeting agents, like for example, Paclitaxel, owing to lower dose-limiting toxicities in non-transformed, proliferative cells. Intriguingly, the Augmin/HAUS complex activity was recently reconstituted *in vitro* in Xenopus egg extracts [53]. It is possible that this assay could be further refined for small molecule inhibitor screens.

Another important question in the context of patient tumors is whether targeting HAUS complex activity will differentially affect GSCs, non-GSCs (i.e., GSC-derived cells) and/or also brain infiltrating tumor cells. Given that all of these cells will share similar features (e.g., oncogenic drivers, levels of aneuploidy, etc) to our HAUS-sensitive GSCs reported here, it seems likely that each population would be sensitive as long as they are traversing mitosis. Taken together, our results reveal a novel regulatory relationship between ZNF131 and the Augmin/HAUS complex that promotes mitotic spindle integrity and cancer cell viability, and suggest that partial inhibition of the Augmin/HAUS complex may represent a new therapeutic strategy for brain tumors.

## MATERIALS AND METHODS

### GSC and NPC cell culture

GSC and NPC lines were grown in N2B27 neural basal media (StemCell Technologies) supplemented with EGF and FGF-2 (20ng/mL each) (Peprotech) on laminin (Sigma) coated polystyrene plates and passaged according to Pollard et al. [8]. GSC isolates were provided by Drs. Steven Pollard (G166, G179), Jeongwu Lee (0131, 0827), Do-Hyun Nam (025T, 578T), and Xiao-Nan Li (1502). NPCs were provided by Drs. Steven Pollard (CB660, U5) and Do-Hyun Nam (779TL) or purchased from Millipore (Vm, Cx). All isolates were validated for over multiple passages for SOX2 and NES expression by either immunofluorescence and/or RNA-sequencing (except for 779TL).

### RNAi

ShRNAs were obtained from Open Biosystems (Huntsville, AL). Target sequences for shRNAs are as follows: ZNF131-1 (V3LHS_322783), ZNF131-2 (V3LHS_322786), ZNF131-3 (V3LHS_322787), HAUS5-1 (V3LHS_373636), HAUS5-2 (V3LHS_373639) and KIF11, CDS:571, AAGAGAGGAGTGATAATTA. ShRNA combo vectors were constructed using the pGIPZ-shHAUS5 vector (for Supplementary Figure S4). The second shRNA (shHAUS2 or shHAUS4) was fused after the HAUS5 hairpin using gibson assembly to make double hairpins construct. The knockdown efficiency of each individual gene was determined by RT-qPCR. The out growth assay was performed using CB660 cells as described below. For virus production pGIPZ-shRNA plasmids were transfected into 293T cells along with psPAX and pMD2.G packaging plasmid to produce lentivirus. Cells were infected at MOI < 1 and selected puromycin (2-4μg/ml) for 2-4 days.

### Lentiviral-mediated ORF expression

RNA was extracted from CB660 cells and reverse transcribed to prepare cDNA library, using SuperScript III RT-PCR kit (Invitrogen). ZNF131 and HAUS5 were PCR amplizied from this cDNA library and cloned into pGUF and pGUM lentiviral expression vectors, which create N-terminal FLAG tag or mCherry fusions (sequence and maps of these vectors are available upon request). We follow above protocol to prepare virus. Expression of each construct was determined by western blot.

### Growth assays

For outgrowth assays, post-selection, shRNA transduced cells were harvested, counted, and plated onto a 96-well plate. After 7 days under standard growth conditions, cell proliferative rate was measured using Alamar blue reagent (Invitrogen).

### Limiting dilution assays

Cells were infected with shRNA virus for 48 hours followed by selection with puromycin for 72 hours (Day 0). Cells were detached from their respective plate, dissociated into single-cell suspensions, counted, and then plated into non-tissue culture treated 96-well plates not coated with laminin with various seeding densities (0.125-256 cells per well, 10 wells per seeding density). Cells were incubated at 37°C for 3 weeks and fed with 10X EGF and FGF-2 neural stem cell expansion media every 3-4 days. At the time of quantification, each well was examined for the formation of tumor spheres.

### Western blots

Western Blots were carried out using standard laboratory practices, except that a modified RIPA buffer was used for protein extraction (150mM NaCl, 50mM Tris, 2mM MgCl2, .1% SDS, .4% DOC, .4% Triton-X 100, 2mM DTT, and complete protease inhibitors (Roche)) followed by a 15 min digestion with 125U of Benzonase (Merck) at RT. The following antibodies were used for detection: Tubulin (1:1000, Cell Signaling) and cleaved Parp (1:1000, Cell Signaling). An Odyssey infrared imaging system was used to visualize blots (Li-cor) following manufacture's instruction.

### Gene expression (RNA-seq)

Cells were lysed with Trizol reagent (Life Technologies), and RNA was extracted according to manufacture instructions (Life Technologies). Total RNA integrity was checked using an Agilent 2200 TapeStation (Agilent Technologies, Inc., Santa Clara, CA) and quantified using a Trinean DropSense96 spectrophotometer (Caliper Life Sciences, Hopkinton, MA). RNA-seq libraries were prepared from total RNA using the TruSeq RNA Sample Prep Kit (Illumina, Inc., San Diego, CA, USA) and libraries size distributions were validated using an Agilent 2200 TapeStation (Agilent Technologies, Santa Clara, CA, USA). Additional library QC, blending of pooled indexed libraries, and cluster optimization was performed using Life Technologies’ Invitrogen Qubit^®^ 2.0 Fluorometer (Life Technologies-Invitrogen, Carlsbad, CA, USA). RNA-seq libraries were pooled and clustered onto a flow cell lane using an Illumina cBot. Sequencing was performed using an Illumina HiSeq 2000 instrument with a read length of 99 bp. Reads of low quality were discarded prior to alignment to the reference genome (UCSC hg19 assembly) using TopHat v2.0.12 [54]. Counts were generated from TopHat alignments for each gene using the Python package HTSeq v0.6.1 [55]. Genes with counts above threshold equal to at least the number of samples in the smallest group were retained, prior to identification of differentially expressed genes using the Bioconductor package edgeR v3.6.8 [56]. A false discovery rate (FDR) method was employed to correct for multiple testing. This output is contained in Supplementary Table S2. All RNA-seq data including raw data will be deposited on GEO.

### RT-qPCR

Quantitect RT-qPCR primer sets and QuantiFast SYBR Green PCR Kits (Qiagen) were used according to the manufacturer's instructions with the ABI Prism 7900 sequence detection system (Genomics Resource, FHCRC). Relative transcript abundance was analyzed using 2−ΔΔCt method. TRIZOL (Invitrogen) extraction was used to collect total RNA from cells.

### ChIP-qPCR

Cells were cross-linked with 1% formaldehyde and sonicated to achieve chromosome fragments of 200-400 bp. ChIP was performed using antibodies directed against RNA Polymerase II (Sigma) according to manufactures protocol (Millipore). qPCR was then carried out as described above, using the primers targeting +1K, −1K, or −6K relative to the *HAUS5* transcription start site (Qiagen).

### Immunofluorescence

Cells were grown on sterile, acid washed coverslips in 35mm cell culture dishes. Cells were rinsed with PHEM (60mM PIPES, 25nM HEPES, 10mM EGTA, 4mM MgSO_4_) and either immediately treated with 4% paraformaldehyde for 20 minutes at room temperature, or for phosphorylation specific antibodies, treated with lysis buffer (PHEM+1.0%Triton X-100) for 5 minutes at 37°C and then PFA fixed for 20 minutes at room temperature. Fixed cells were washed, blocked for 1 hour at room temperature in PHEM+10% boiled donkey serum (BDS). Primary antibodies were diluted in PHEM+5% BDS and incubated for 16 hours at 4°C. Coverslips were washed, then incubated with secondary antibodies conjugated to fluorescent dyes (Jackson ImmunoReserach Laboratories) again diluted in PHEM+5% BDS for 45 minutes at room temperature. Coverslips were washed and stained with 2 ng/mL 4′,6-diamidino-2-phenylindole (DAPI) diluted in PHEM then mounted onto microscope slides in an anti-fade solution containing 90% glycerol and 0.5%*N*-propyl gallate.

Commercial antibodies used: α-Tubulin (Sigma, T-6199); acetylated α-Tubulin (Sigma, T-7451); γ-Tubulin (Sigma, T-5326); PCM1 (Cell Signaling, 5213) and HAUS6 (gift from Laurence Pelletier's lab).

### Image acquisition and analysis

Fixed-cell images were acquired using a DeltaVision PersonalDV Imaging System (Applied Precision/GE) on a Photometrics CoolSnap HQ2 camera (Roper Scientific) and a 60x/1.42NA Planapochromat DIC oil immersion lens (Olympus). All immunofluorescence images were collected as z-stacks at 0.2 μm intervals. Kinetochore integrated pixel intensity values were measured on deconvolved images with SoftWorx software (Applied Precision) applying background correction. To measure centrosome volume, the cells were stained with PCM1 antibody and then imaged using Deltavision imaging system. The centrosome volume was determined by centrosomal fluorescence intensity from deconvolved 3D images using Velocity software. An arbitrary threshold was applied to remove the particles with nonspecific staining [34].

### Xenografts

GSC-0131 cells were infected with pGIPZ-shRNA virus and selected for 3 days in puromycin (2μg/mL), such that > 80% of cells were GFP+. Cells were then harvested using Accutase (Sigma), counted, resuspended in an appropriate volume of culture media, and kept on ice prior to immediate transplantation. NOD-scid IL2Rgammanull mice (Jackson Labs #005557) were anesthetized by IP injection of 0.2ml/10 grams 1.25% Avertin Solution and kept at 37°C. A small bore hole was made in the skull using a hand drill with a Meisinger #009 steel burr bit (Hager & Meisinger GmbH). 2×10^5 cells were slowly injected by pipet into the right frontal cortex approximately 2mm rostral to Bregma, 2mm lateral and 3mm deep through a 0.2-10ul disposable sterile aerosol barrier tip (Fisher Scientific #02-707-30). The burr hole was closed using SURGIFOAM (Johnson & Johnson) and the skin rejoined using TISSUMEND II (Veterinary Product Laboratories, Phoenix AZ). Seven weeks after initial transplantation mice were injected intravenously with 50μl of 40μM Chlorotoxin: Cy5.5 conjugate [57] 2 hours prior to sacrifice by carbon dioxide inhalation. The brain and tumor were removed from the skull and imaged for Cy5.5 and GFP fluorescence using the Xenogen IVIS Spectrum imaging system (Caliper Life Sciences).

## SUPPLEMENTARY MATERIAL FIGURES AND TABLES







## References

[1] American Cancer Society (2010). American Cancer Society: Cancer Facts and Figures.

[2] Stupp R, Mason WP, van den Bent MJ, Weller M, Fisher B, Taphoorn MJ, Belanger K, Brandes AA, Marosi C, Bogdahn U, Curschmann J, Janzer RC, Ludwin SK (2005). Radiotherapy plus concomitant and adjuvant temozolomide for glioblastoma. N Engl J Med.

[3] Hemmati HD, Nakano I, Lazareff JA, Masterman-Smith M, Geschwind DH, Bronner-Fraser M, Kornblum HI (2003). Cancerous stem cells can arise from pediatric brain tumors. Proc Natl Acad Sci U S A.

[4] Singh SK, Clarke ID, Terasaki M, Bonn VE, Hawkins C, Squire J, Dirks PB (2003). Identification of a cancer stem cell in human brain tumors. Cancer Res.

[5] Singh SK, Hawkins C, Clarke ID, Squire JA, Bayani J, Hide T, Henkelman RM, Cusimano MD, Dirks PB (2004). Identification of human brain tumour initiating cells. Nature.

[6] Galli R, Binda E, Orfanelli U, Cipelletti B, Gritti A, De Vitis S, Fiocco R, Foroni C, Dimeco F, Vescovi A (2004). Isolation and characterization of tumorigenic, stem-like neural precursors from human glioblastoma. Cancer Res.

[7] Lee J, Kotliarova S, Kotliarov Y, Li A, Su Q, Donin NM, Pastorino S, Purow BW, Christopher N, Zhang W, Park JK, Fine HA (2006). Tumor stem cells derived from glioblastomas cultured in bFGF and EGF more closely mirror the phenotype and genotype of primary tumors than do serum-cultured cell lines. Cancer Cell.

[8] Pollard SM, Yoshikawa K, Clarke ID, Danovi D, Stricker S, Russell R, Bayani J, Head R, Lee M, Bernstein M, Squire JA, Smith A, Dirks P (2009). Glioma stem cell lines expanded in adherent culture have tumor-specific phenotypes and are suitable for chemical and genetic screens. Cell Stem Cell.

[9] Hubert CG, Bradley RK, Ding Y, Toledo CM, Herman J, Skutt-Kakaria K, Girard EJ, Davison J, Berndt J, Corrin P, Hardcastle J, Basom R, Delrow JJ (2013). Genome-wide RNAi screens in human brain tumor isolates reveal a novel viability requirement for PHF5A. Genes Dev.

[10] Ding Y, Hubert CG, Herman J, Corrin P, Toledo CM, Skutt-Kakaria K, Vazquez J, Basom R, Zhang B, Risler JK, Pollard SM, Nam DH, Delrow JJ (2013). Cancer-Specific requirement for BUB1B/BUBR1 in human brain tumor isolates and genetically transformed cells. Cancer Discov.

[11] Toledo CM, Herman JA, Olsen JB, Ding Y, Corrin P, Girard EJ, Olson JM, Emili A, DeLuca JG, Paddison PJ (2014). BuGZ is required for Bub3 stability, Bub1 kinetochore function, and chromosome alignment. Dev Cell.

[12] Sun Y, Pollard S, Conti L, Toselli M, Biella G, Parkin G, Willatt L, Falk A, Cattaneo E, Smith A (2008). Long-term tripotent differentiation capacity of human neural stem (NS) cells in adherent culture. Mol Cell Neurosci.

[13] Donaldson NS, Nordgaard CL, Pierre CC, Kelly KF, Robinson SC, Swystun L, Henriquez R, Graham M, Daniel JM (2010). Kaiso regulates Znf131-mediated transcriptional activation. Experimental cell research.

[14] Tommerup N, Vissing H (1995). Isolation and fine mapping of 16 novel human zinc finger-encoding cDNAs identify putative candidate genes for developmental and malignant disorders. Genomics.

[15] Trappe R, Buddenberg P, Uedelhoven J, Glaser B, Buck A, Engel W, Burfeind P (2002). The murine BTB/POZ zinc finger gene Znf131: predominant expression in the developing central nervous system, in adult brain, testis, and thymus. Biochem Biophys Res Commun.

[16] Han X, Guo J, Deng W, Zhang C, Du P, Shi T, Ma D (2008). High-throughput cell-based screening reveals a role for ZNF131 as a repressor of ERalpha signaling. BMC Genomics.

[17] Sawin KE, LeGuellec K, Philippe M, Mitchison TJ (1992). Mitotic spindle organization by a plus-end-directed microtubule motor. Nature.

[18] Venere M, Horbinski C, Crish JF, Jin X, Vasanji A, Major J, Burrows AC, Chang C, Prokop J, Wu Q, Sims PA, Canoll P, Summers MK (2015). The mitotic kinesin KIF11 is a driver of invasion, proliferation, and self-renewal in glioblastoma. Sci Transl Med.

[19] Donaldson NS, Nordgaard CL, Pierre CC, Kelly KF, Robinson SC, Swystun L, Henriquez R, Graham M, Daniel JM (2010). Kaiso regulates Znf131-mediated transcriptional activation. Exp Cell Res.

[20] Oh Y, Chung KC (2012). Small ubiquitin-like modifier (SUMO) modification of zinc finger protein 131 potentiates its negative effect on estrogen signaling. J Biol Chem.

[21] Blattler A, Yao L, Wang Y, Ye Z, Jin VX, Farnham PJ (2013). ZBTB33 binds unmethylated regions of the genome associated with actively expressed genes. Epigenetics Chromatin.

[22] Parisi M, Glass I, Pagon RA, Adam MP, Ardinger HH, Wallace SE, Amemiya A, Bean LH, Bird TD, Dolan CR, Fong CT, Smith RJ, Stephens K (1993-2017). Joubert Syndrome and Related Disorders. Gene Reviews.

[23] Sharma N, Berbari NF, Yoder BK (2008). Ciliary dysfunction in developmental abnormalities and diseases. Curr Top Dev Biol.

[24] Firat-Karalar EN, Sante J, Elliott S, Stearns T (2014). Proteomic analysis of mammalian sperm cells identifies new components of the centrosome. J Cell Sci.

[25] Uehara R, Nozawa RS, Tomioka A, Petry S, Vale RD, Obuse C, Goshima G (2009). The augmin complex plays a critical role in spindle microtubule generation for mitotic progression and cytokinesis in human cells. Proc Natl Acad Sci U S A.

[26] Lawo S, Bashkurov M, Mullin M, Ferreria MG, Kittler R, Habermann B, Tagliaferro A, Poser I, Hutchins JR, Hegemann B, Pinchev D, Buchholz F, Peters JM (2009). HAUS, the 8-subunit human Augmin complex, regulates centrosome and spindle integrity. Curr Biol.

[27] Goshima G, Mayer M, Zhang N, Stuurman N, Vale RD (2008). Augmin: a protein complex required for centrosome-independent microtubule generation within the spindle. J Cell Biol.

[28] Nigg EA, Stearns T (2011). The centrosome cycle: Centriole biogenesis, duplication and inherent asymmetries. Nat Cell Biol.

[29] Pollak J, Rai KG, Funk CC, Arora S, Lee E, Zhu J, Price ND, Paddison PJ, Ramirez JM, Rostomily RC (2017). Ion channel expression patterns in glioblastoma stem cells with functional and therapeutic implications for malignancy. PLoS One.

[30] Kollman JM, Merdes A, Mourey L, Agard DA (2011). Microtubule nucleation by gamma-tubulin complexes. Nat Rev Mol Cell Biol.

[31] Khodjakov A, Rieder CL (1999). The sudden recruitment of gamma-tubulin to the centrosome at the onset of mitosis and its dynamic exchange throughout the cell cycle, do not require microtubules. J Cell Biol.

[32] Dictenberg JB, Zimmerman W, Sparks CA, Young A, Vidair C, Zheng Y, Carrington W, Fay FS, Doxsey SJ (1998). Pericentrin and gamma-tubulin form a protein complex and are organized into a novel lattice at the centrosome. J Cell Biol.

[33] Decker M, Jaensch S, Pozniakovsky A, Zinke A, O'Connell KF, Zachariae W, Myers E, Hyman AA (2011). Limiting amounts of centrosome material set centrosome size in C. elegans embryos. Curr Biol.

[34] Zyss D, Gergely F (2009). Centrosome function in cancer: guilty or innocent?. Trends Cell Biol.

[35] Reynolds BA, Weiss S (1992). Generation of neurons and astrocytes from isolated cells of the adult mammalian central nervous system. Science.

[36] Hubner NC, Wang LH, Kaulich M, Descombes P, Poser I, Nigg EA (2010). Re-examination of siRNA specificity questions role of PICH and Tao1 in the spindle checkpoint and identifies Mad2 as a sensitive target for small RNAs. Chromosoma.

[37] Lingle WL, Lutz WH, Ingle JN, Maihle NJ, Salisbury JL (1998). Centrosome hypertrophy in human breast tumors: implications for genomic stability and cell polarity. Proc Natl Acad Sci U S A.

[38] Neben K, Giesecke C, Schweizer S, Ho AD, Kramer A (2003). Centrosome aberrations in acute myeloid leukemia are correlated with cytogenetic risk profile. Blood.

[39] Kramer A, Schweizer S, Neben K, Giesecke C, Kalla J, Katzenberger T, Benner A, Muller-Hermelink HK, Ho AD, Ott G (2003). Centrosome aberrations as a possible mechanism for chromosomal instability in non-Hodgkin's lymphoma. Leukemia.

[40] Jung CK, Jung JH, Lee KY, Kang CS, Kim M, Ko YH, Oh CS (2007). Centrosome abnormalities in non-small cell lung cancer: correlations with DNA aneuploidy and expression of cell cycle regulatory proteins. Pathol Res Pract.

[41] Sato N, Mizumoto K, Nakamura M, Nakamura K, Kusumoto M, Niiyama H, Ogawa T, Tanaka M (1999). Centrosome abnormalities in pancreatic ductal carcinoma. Clin Cancer Res.

[42] Kwon M, Godinho SA, Chandhok NS, Ganem NJ, Azioune A, Thery M, Pellman D (2008). Mechanisms to suppress multipolar divisions in cancer cells with extra centrosomes. Genes Dev.

[43] Haren L, Merdes A (2002). Direct binding of NuMA to tubulin is mediated by a novel sequence motif in the tail domain that bundles and stabilizes microtubules. J Cell Sci.

[44] Gaglio T, Saredi A, Compton DA (1995). NuMA is required for the organization of microtubules into aster-like mitotic arrays. J Cell Biol.

[45] Maiato H, Logarinho E (2014). Mitotic spindle multipolarity without centrosome amplification. Nat Cell Biol.

[46] Herman JA, Toledo CM, Olson JM, DeLuca JG, Paddison PJ (2015). Molecular pathways: regulation and targeting of kinetochore-microtubule attachment in cancer. Clin Cancer Res.

[47] Vartanian A, Singh SK, Agnihotri S, Jalali S, Burrell K, Aldape KD, Zadeh G (2014). GBM's multifaceted landscape: highlighting regional and microenvironmental heterogeneity. Neuro Oncol.

[48] Patel AP, Tirosh I, Trombetta JJ, Shalek AK, Gillespie SM, Wakimoto H, Cahill DP, Nahed BV, Curry WT, Martuza RL, Louis DN, Rozenblatt-Rosen O, Suva ML (2014). Single-cell RNA-seq highlights intratumoral heterogeneity in primary glioblastoma. Science.

[49] Sottoriva A, Spiteri I, Piccirillo SG, Touloumis A, Collins VP, Marioni JC, Curtis C, Watts C, Tavare S (2013). Intratumor heterogeneity in human glioblastoma reflects cancer evolutionary dynamics. Proc Natl Acad Sci U S A.

[50] Toledo CM, Ding Y, Hoellerbauer P, Davis RJ, Basom R, Girard EJ, Lee E, Corrin P, Hart T, Bolouri H, Davison J, Zhang Q, Hardcastle J (2015). Genome-wide CRISPR-Cas9 Screens Reveal Loss of Redundancy between PKMYT1 and WEE1 in Glioblastoma Stem-like Cells. Cell Rep.

[51] Shalem O, Sanjana NE, Hartenian E, Shi X, Scott DA, Mikkelsen TS, Heckl D, Ebert BL, Root DE, Doench JG, Zhang F (2014). Genome-scale CRISPR-Cas9 knockout screening in human cells. Science.

[52] Wang T, Wei JJ, Sabatini DM, Lander ES (2014). Genetic screens in human cells using the CRISPR-Cas9 system. Science.

[53] Hsia KC, Wilson-Kubalek EM, Dottore A, Hao Q, Tsai KL, Forth S, Shimamoto Y, Milligan RA, Kapoor TM (2014). Reconstitution of the augmin complex provides insights into its architecture and function. Nat Cell Biol.

[54] Trapnell C, Pachter L, Salzberg SL (2009). TopHat: discovering splice junctions with RNA-Seq. Bioinformatics.

[55] Anders S, Pyl PT, Huber W (2015). HTSeq--a Python framework to work with high-throughput sequencing data. Bioinformatics.

[56] Robinson MD, McCarthy DJ, Smyth GK (2010). edgeR: a Bioconductor package for differential expression analysis of digital gene expression data. Bioinformatics.

[57] Veiseh M, Gabikian P, Bahrami SB, Veiseh O, Zhang M, Hackman RC, Ravanpay AC, Stroud MR, Kusuma Y, Hansen SJ, Kwok D, Munoz NM, Sze RW (2007). Tumor paint: a chlorotoxin: Cy5.5 bioconjugate for intraoperative visualization of cancer foci. Cancer Res.

