# Supplementary material for: ZNF131 suppresses centrosome fragmentation in glioblastoma stem-like cells through regulation of HAUS5
Source: Oncotarget. 2017 May 24;8(30):48545–62. doi: 10.18632/oncotarget.18153 (PMC5564707; doi:10.18632/oncotarget.18153)
Supplement: Supplementary file 1 [file oncotarget-08-48545-s001.pdf]

ZNF131 suppresses centrosome fragmentation in glioblastoma stem-like cells through regulation of HAUS5

Supplementary Material

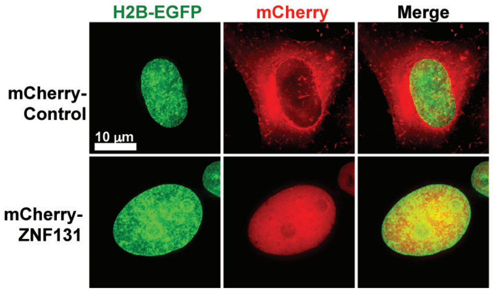

Supplementary Figure S1. Localizaition of mCherry-ZNF131 to the nucleus of GSC-0131 cells. The mCherry was fused to the N-termincal of the ORF in the pGUM lentiviral expression vector.. GSC-0131 cells were infected and ~2 days after selection, the expression of fusion protein was visualised using Delta vision imaging system.

A

	PMID	Name	pValue
♋	26638075	A Dynamic Protein Interaction Landscape of the Human Centrosome-Cilium Interface.	1.60E-11
	25921289	Temporal proteomics of NGF-TrkA signaling…	3.58E-09
	17081983	Global, in vivo, and site-specific phosphorylation dynamics in signaling networks.	1.96E-08
	19946888	Defining the membrane proteome of NK cells.	8.80E-08
	24711643	Identifying biological pathways that underlie primordial short stature using network analysis.	2.66E-07
	20467437	Direct interaction between hnRNP-M and CDC5L/PLRG1 proteins affects alternative splice site choice.	3.63E-07
	25609649	Proteomic analyses reveal distinct chromatin-associated and soluble transcription factor complexes.	5.74E-07
	25281560	Interactome mapping of chromatin-associated protein complexes.	7.16E-07
♌	20301500	Joubert Syndrome and Related Disorders	1.66E-06
♌	21633164	Mutations in KIF7 link Joubert syndrome with Sonic Hedgehog signaling and microtubule dynamics.	3.95E-06

B

***ZNF131* regulated mRNAs**

	GSC-0131	GSC-G166	GSC-0827
♋*CCDC113*	-4.41	-1.88	-2.91
♂♋*HAUS5*	-2.19	-1.21	-1.43
ZNF131	-1.69	-1.14	-1.20
♌♋*NPHP1*	-1.69	-1.05	-0.57
♋*MIF4GD*	-1.53	-0.65	-0.89
♌♋*TMEM237*	-1.19	-0.66	-1.15
♋*LCLAT1*	-0.80	-0.32	-0.75
♌♋*KIF7*	-0.77	-0.78	-1.91
♋*AP2B1*	-0.75	-0.22	-0.23
♋*LBR*	-0.55	-0.17	-0.21
♋*C21orf2*	-0.53	-0.61	-0.44
♋*NDUFV1*	-0.52	-0.26	-0.23
♋*TMEM230*	-0.41	-0.30	-0.20
♋*ARF5*	-0.37	-0.23	-0.34
♋*SMARCAD1*	-0.32	-0.22	-0.24
♋*UXT*	-0.23	-0.22	-0.26
♋*NUP155*	0.43	0.31	0.21
♋*SMPD4*	0.48	0.36	0.21
♋*LONP1*	0.65	0.45	0.19

♋Centrosome-Cilium Interface
♌Joubert Syndrome and Related Disorders
♂Microtubule nucleation

Supplementary Figure S2. Gene Set Enrichment Analysis for genes whose expression is changed by ZNF131 knockdown in GSCs. (A) Enriched gene sets arising from specific publications (ToppGene Suite; reference #22). **(B)** Gene expression data of the genes scoring in "Centrosome-Cilium Interface" and "Joubert Syndrome and Related Disorders" from (A). Values are log2-fold-change of shZNF131 versus shControl samples. Experiments were performed according to Methods.

Figure S3

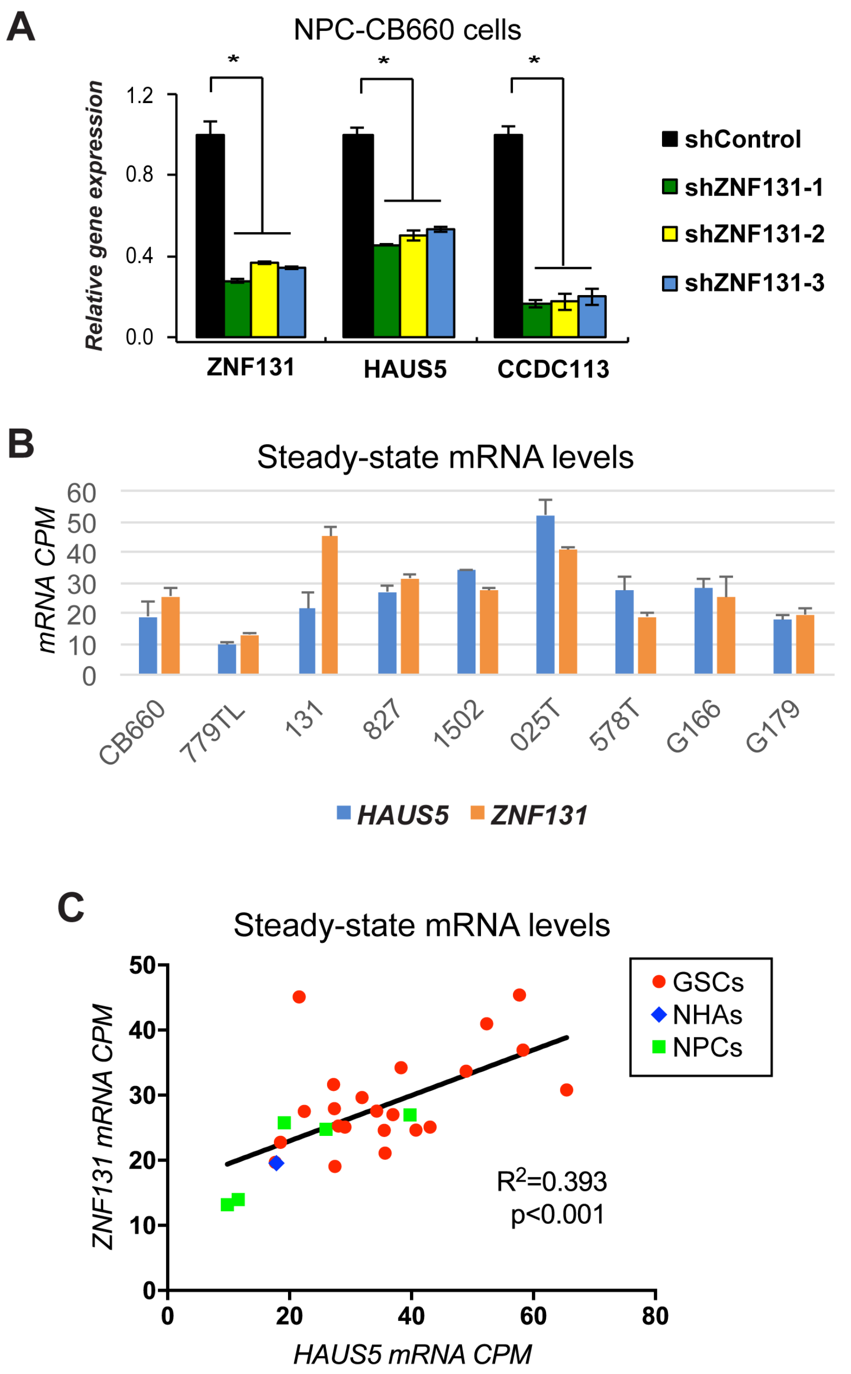

Supplementary Figure S3. Further examination of reatlionship between *ZNF131* and HAUS5 gene expression. (A) RT-qPCR confirmation of shZNF131-depedent downregulation of ZNF131, HAUS5, and CCDC113 in NPCs. Experiments were performed as in Figure 2. **(B)** RNA-seq results for HAUS5 and ZNF131 for NPCs and GSCs shown in Figure 1C. NPC-CB660 and NPC-779TL are resistent, while all GSCs were shZNF131 sensitive. **(C)** More extensive RNA-seq comparisons of 28 different cell isolates, including NPCs, normal human astrocytes (NHA) and GSCs. RNA-seq data is from reference # . CPM = counts per million reads mapped.

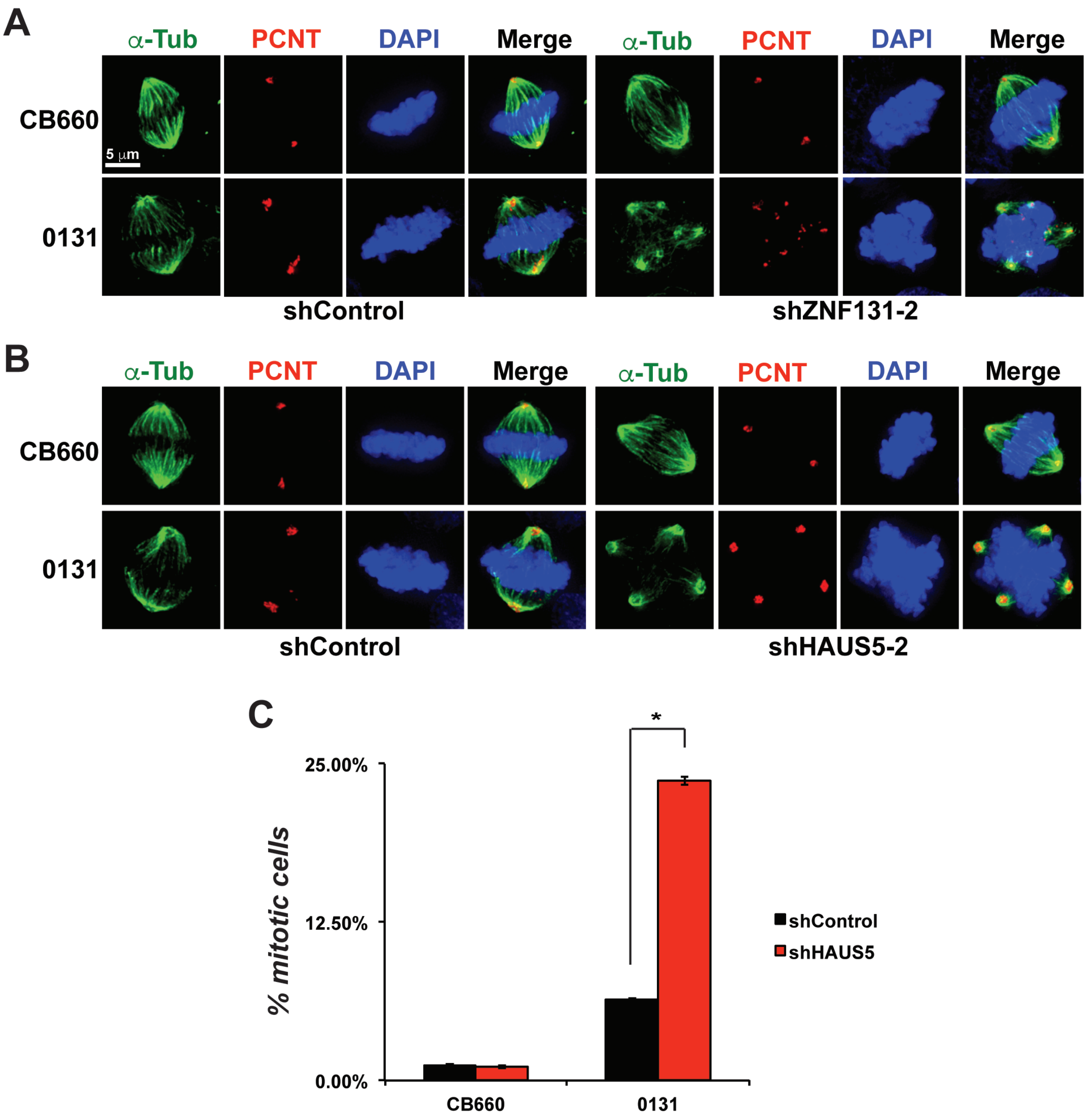

Supplementary Figure S4. *ZNF131* and *HAUS5* kd cause centrosome fragmentation and multi-polar spindle formation in GSC-0131 but not NPC-CB660 cells. (A) & **(B)** Representative images of of NPC-CB660 and and GSC-0131 cells after knockdown of *ZNF131* or *HAUS5*. Cells were were stained for pericentrin and tubulin 48hrs after selection for LV-shRNAs acccoriding to Methods. Centrosome size was used as a surrogate for mutlipolarity in these experiments, which showed dramatic differences (Fig. 5). **(C)** Mitotic index dramatically increases in GSC-0131 but not NPC-CB660 after *HAUS5* kd, similar to ZNF131 kd (Fig. 1). *indicates p-value<.001.

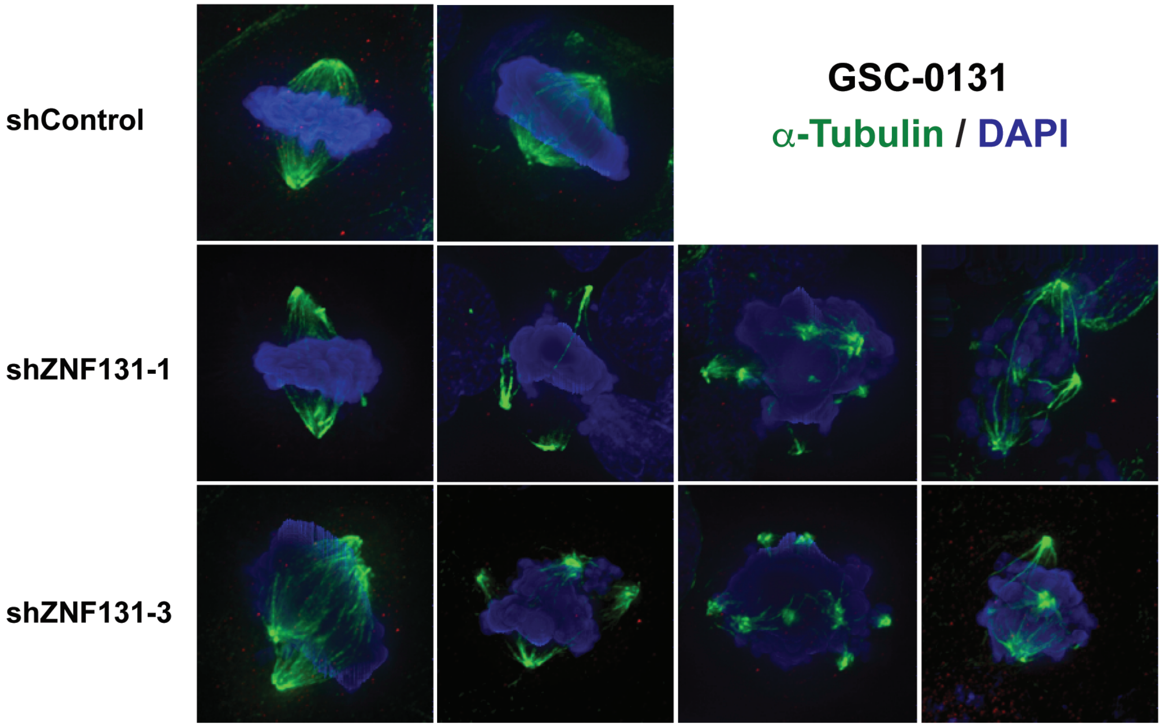

Supplementary Figure S5. Multiple *ZNF131* shRNAs cause centrosome fragmentation and multipolar spindle formation in GSC-0131 cells. Representative images of GSC-0131 cells after knockdown of *ZNF131* using two additonal shRNAs, which were validated in Figure 1 for knockdown of gene expression and viability loss in GSCs. Cells were were stained for tubulin 48hrs after selection for LV-shRNAs accoriding to Methods.

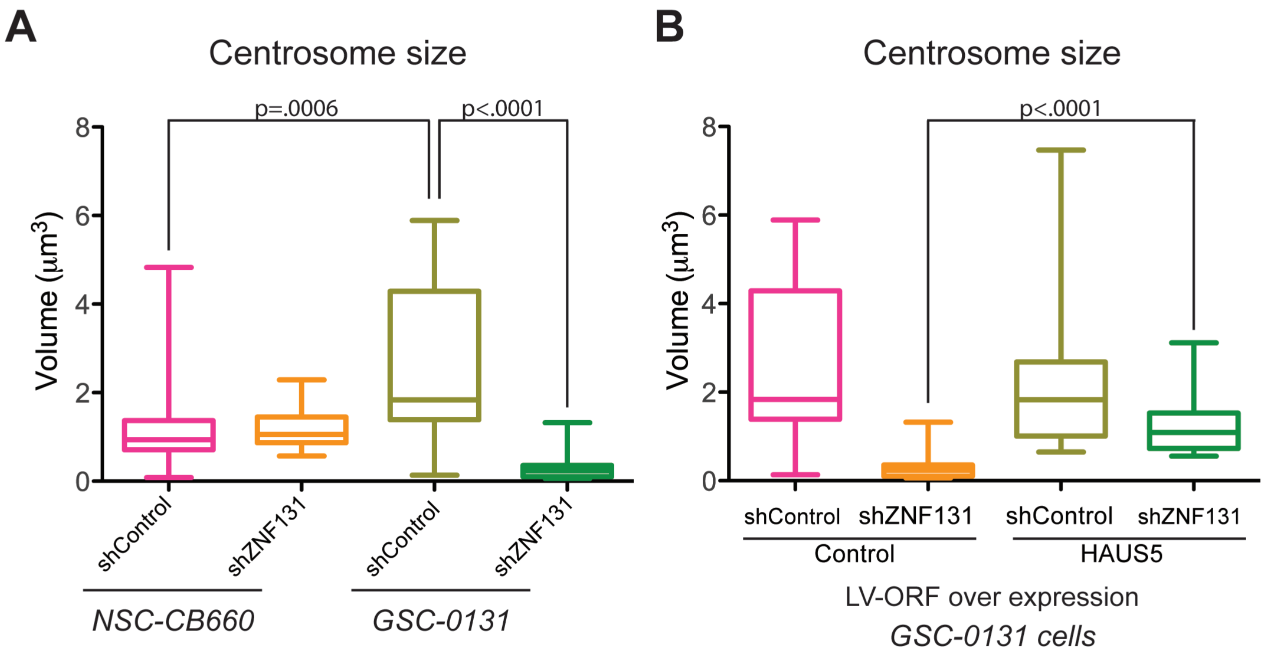

Supplementary Figure S6. Measurement of centrosome size in GSCs and NPCs after ZNF131 knockdown and complementation with HAUS5. (A) ZNF131 kd causes reduction in centrosome size in GSCs but not NPCs. Centrosome size was measured using pericentrin staining as outlined in Methods. **(B)** Ectopic expression of HAUS5 ORF complements loss of centrosome size in GSC-0131 cells. Cells were first infected with LV containing control or HAUS5 ORF followed by LV-shRNA and assayed 48hrs after selection (n=31 for each group).

Gene expression of Augmin/HAUS complex subunits (CPM)

	CB660	779TL	131	827	025T	1502	578T	G166	G179
HAUS1	21.25	8.13	32.67	66.77	39.89	30.91	24.60	29.46	21.74
HAUS2	31.58	14.58	49.50	53.93	49.26	50.67	42.67	52.85	26.07
HAUS3	17.49	10.15	21.63	41.04	32.97	31.96	20.38	29.69	25.36
HAUS4	22.03	8.95	25.33	83.22	11.60	16.77	17.21	42.39	27.85
HAUS5	19.12	9.79	21.57	27.21	52.33	34.27	27.42	28.03	17.68
HAUS6	51.02	24.30	63.44	197.12	245.90	94.85	109.19	86.52	39.81
HAUS7	6.61	10.83	29.81	39.00	22.54	23.44	17.26	18.99	17.60
HAUS8	5.90	2.04	17.95	25.79	17.81	17.35	16.99	13.06	13.96

Supplementary Figure S7. Augmin/HAUS complex subunit gene expression. RNA-seq results for HAUS5 and ZNF131 for NPCs and GSCs shown in Figure 1C. NPC-CB660 and NPC-779TL are resistent, while all GSCs were shZNF131 sensitive. RNA-seq data is from reference #29. Values are counts per million reads mapped.
